# Assessing Outcomes in HIV Prevention and Treatment Programs With Female Sex Workers and Men Who Have Sex With Men: Expanded Polling Booth Survey Protocol

**DOI:** 10.2196/54313

**Published:** 2024-06-19

**Authors:** Parinita Bhattacharjee, Leigh M McClarty, Joshua Kimani, Shajy Isac, Rhoda Wanjiru Kabuti, Antony Kinyua, Jaffred Karakaja Okoyana, Virjinia Njeri Ndukuyu, Helgar Musyoki, Anthony Kiplagat, Peter Arimi, Souradet Shaw, Faran Emmanuel, Monica Gandhi, Marissa Becker, James Blanchard

**Affiliations:** 1 Institute for Global Public Health University of Manitoba Winnipeg, MB Canada; 2 Partners for Health and Development in Africa Nairobi Kenya; 3 India Health Action Trust Delhi India; 4 Sex Workers Outreach Program Clinic Nairobi Kenya; 5 The Global Fund to Fight AIDS, Tuberculosis and Malaria Nairobi Kenya; 6 Nairobi County Health Department Nairobi Kenya; 7 School of Medicine University of California at San Francisco San Francisco, CA United States

**Keywords:** female sex workers, men who have sex with men, Kenya, polling booth survey, program science, HIV prevention, outcome assessment

## Abstract

**Background:**

Assessing HIV outcomes in key population prevention programs is a crucial component of the program cycle, as it facilitates improved planning and monitoring of anticipated results. The Joint United Nations Programme on HIV and AIDS recommends using simple, rapid methods to routinely measure granular and differentiated program outcomes for key populations. Following a program science approach, Partners for Health and Development in Africa, in partnership with the Nairobi County Government and the University of Manitoba, aims to conduct an outcome assessment using a novel, expanded polling booth survey (ePBS) method with female sex workers and men who have sex with men in Nairobi County, Kenya.

**Objective:**

This study aims to (1) estimate the incidence and prevalence of HIV; (2) assess biomedical, behavioral, and structural outcomes; and (3) understand barriers contributing to gaps in access and use of available prevention and treatment services among female sex workers and men who have sex with men in Nairobi.

**Methods:**

The novel ePBS approach employs complementary data collection methods, expanding upon the traditional polling booth survey (PBS) method by incorporating additional quantitative, qualitative, and biological data collection components and an improved sampling methodology. Quantitative methods will include (1) PBS, a group interview method in which individuals provide responses through a ballot box in an unlinked and anonymous way, and (2) a behavioral and biological survey (BBS), including a face-to-face individual interview and collection of linked biological samples. Qualitative methods will include focus group discussions. The ePBS study uses a 2-stage, population- and location-based random sampling approach involving the random selection of locations from which random participants are selected at a predetermined time on a randomly selected day. PBS data will be analyzed at the group level, and BBS data will be analyzed at an individual level. Qualitative data will be analyzed thematically.

**Results:**

Data were collected from April to May 2023. The study has enrolled 759 female sex workers (response rate: 759/769, 98.6%) and 398 men who have sex with men (response rate: 398/420, 94.7%). Data cleaning and analyses are ongoing, with a focus on assessing gaps in program coverage and inequities in program outcomes.

**Conclusions:**

The study will generate valuable HIV outcome data to inform program improvement and policy development for Nairobi County’s key population HIV prevention program. This study served as a pilot for the novel ePBS method, which combines PBS, BBS, and focus group discussions to enhance its programmatic utility. The ePBS method holds the potential to fill an acknowledged gap for a rapid, low-cost, and simple method to routinely measure HIV outcomes within programs and inform incremental program improvements through embedded learning processes.

## Introduction

### Background

Kenya has made significant strides in its HIV response, evident by the continuous decline in HIV prevalence among adults aged 15 to 49 years within the general population. HIV prevalence has dropped from a peak of approximately 10% in the mid-1990s to 4.3% in 2021 [1]. An analysis of the HIV epidemic within Kenya [2] highlights substantial heterogeneity. HIV prevalence varies geographically, ranging from 20.1% in Homa Bay County to 0.2% in Mandera and Wajir counties. Notably, HIV prevalence in Kenya is also disproportionally high among female individuals (5.5%) than among male individuals (2.9%) [3]. Furthermore, compared to the general Kenyan population, HIV prevalence is significantly higher among key populations, namely, female sex workers at 29%, men who have sex with men at 18.9%, people who inject drugs at 18%, and transgender people at 40% [4,5]. This underscores the critical importance of properly scaled and tailored interventions to address the HIV epidemic within key populations in the country [4].

In 2020, a nationwide mapping and population size estimation exercise estimated that there were 197,096 female sex workers and 61,650 men who have sex with men living in Kenya (National AIDSSTI Control Programme, unpublished data, December 2020). Approximately 20% of key populations are concentrated in Nairobi County, the largest of the 47 counties in Kenya [6]. Findings from a 2017 national study underscore the high prevalence of HIV among female sex workers and men who have sex with men in Nairobi, with self-reported rates of 22% and 27%, respectively [7]. In the same study, 91% of female sex workers in Nairobi reported using condoms with their last client, while 81% of men who have sex with men reported condom use during last anal sex [7]. Furthermore, peer educators played a significant role in outreach efforts, with 88% of female sex workers and 76% of men who have sex with men reporting contact with a peer educator within the last 3 months. The majority (82%) of both groups had undergone HIV testing within the last 3 months, while 22% of female sex workers and 27% of men who have sex with men reported that they were living with HIV. However, only 65% of female sex workers and 68% of men who have sex with men living with HIV reported being enrolled in antiretroviral therapy programs. Notably, 56% of female sex workers and 18% of men who have sex with men reported experiencing incidents of police violence within the last 6 months, respectively [7].

HIV prevention programs for key populations in Nairobi County are implemented by several partner organizations, all of whom report to the county government and the National Key Population Program, led by the Kenyan Ministry of Health, on a monthly basis (National AIDS and STI Control Programme, unpublished data, September 2023). Having up-to-date, representative, and robust data on key populations is of paramount importance for effective planning, management, and monitoring of programs and interventions aimed at addressing their needs [8]. The most recent population-based behavioral survey conducted among key populations in Nairobi dates back to 2017 [7] and did not include biological data. The absence of recent biobehavioral data among key population groups limits the ability of both county and national programs to understand the current epidemiologic situation, assess the impact of investments in HIV prevention and treatment programs for key populations, and identify gaps in program coverage. The high cost and resource requirements for implementing nationally representative, population-based integrated biobehavioral surveillance surveys factor into the relative infrequency of their deployment [8]. The availability of methods that can efficiently collect timely data among key population groups at appropriate scale and relatively low cost across diverse contexts is extremely limited [9]. Striking a balance between the need for methodological rigor and practical, adaptable approaches that are accessible to countries with diverse income levels is a pressing requirement [10].

### Objectives

Partners for Health and Development in Africa (PHDA), in collaboration with the University of Manitoba and the Nairobi County Government, aims to conduct an outcome assessment study using a novel, expanded polling booth survey (ePBS) method for data collection. This outcome assessment focuses on 2 key populations with whom PHDA works in Nairobi County, Kenya: female sex workers and men who have sex with men. The objectives of the study are to (1) estimate the incidence and prevalence of HIV; (2) assess biomedical (HIV testing, condom use, pre-exposure prophylaxis [PrEP] uptake, and HIV treatment), behavioral (knowledge and risk behavior), and structural outcomes (experiences of violence, stigma, and discrimination); and (3) gain a deeper understanding of barriers that contribute to disparities in accessing and using HIV services among female sex workers and men who have sex with men in Nairobi County, Kenya. Importantly, this study will pilot the feasibility of the novel ePBS method to measure outcomes in key population programs, with the intention of disseminating the approach for scaling up in other key population programs within and outside Kenya.

## Methods

### Study Setting

Nairobi County is the largest of Kenya’s 47 counties by population and has a robust key population program. According to the latest mapping and population size estimation exercise, Nairobi County has the highest concentration of female sex workers (n=39,227) and men who have sex with men (n=15,271) living or working in the country, or both [6]. The county has identified 2032 locations, across its 12 subcounties, where female sex workers solicit clients and 369 locations where men who have sex with men meet their sexual partners [6]. Bars (without lodging) have the highest proportion of female sex workers and men who have sex with men among these locations [6].

Importantly, same-sex sexual practices are criminalized throughout Kenya, and in 2017, Nairobi County implemented a ban on all forms of sex work [11,12]. This has contributed to the elevated levels of violence, stigma, and discrimination experienced by female sex workers and men who have sex with men [7] within the county. There are currently 4 large implementing partners responsible for prevention programs for female sex workers and men who have sex with men in Nairobi, which are funded by the Global Fund to Fight AIDS, Tuberculosis, and Malaria and the US President’s Emergency Plan for AIDS Relief. Among these partners, PHDA implements the largest prevention program in Nairobi and Kenya, serving approximately 34,000 female sex workers and 14,000 men who have sex with men. PHDA reaches these groups across all 17 counties in Kenya through an extensive network of peer educators and 9 Sex Workers Outreach Program (SWOP) clinics. To implement this program, PHDA receives funding from the US President’s Emergency Plan for AIDS Relief and technical support from the University of Manitoba and the University of Maryland, Baltimore. Furthermore, this collaboration involves Nairobi County and the National AIDS and STI Control Programme (NASCOP) [13].

### Program Science Approach

This study adopts a program science approach, which systematically applies scientific theory and empirical knowledge to answer critical programmatic questions that are generated by the program itself [14]. The approach seamlessly integrates the program cycle with research questions, strategically embedding iterative cycles of research and learning within a program, thus facilitating rapid uptake of knowledge and evidence into program operations. Furthermore, the program science approach generates embedded research agendas to address ongoing programmatic challenges [15]. In the context of this study, the research questions and study objectives have evolved from 2 primary programmatic concerns within the Nairobi County’s key population program. The first concern revolves around the absence of recent population-based data to assess program outcomes (biomedical, behavioral, and structural) pertinent to key populations in Nairobi County. The second concern is the lack of a low-cost, reliable, and easily reproducible method capable of routinely evaluating granular and differentiated program outcomes for key populations, assessing and identifying gaps in the program’s coverage, and making programmatic adjustments to address emerging challenges.

Program science emphasizes the importance of routinely identifying, quantifying, examining, and addressing gaps in program coverage, through tools, such as the effective program coverage framework, to reduce inequities in health outcomes and achieve population-level impact [16]. A program science approach underscores the importance of engaging affected communities [17] and actively works toward developing program and policy solutions to public health challenges [18]. As such, this study is conducted by PHDA in partnership with the community advisory board of the SWOP clinics and the Department of Public Health, Nairobi County.

### Research Questions

The study sets out to answer four specific research questions.

1. What is the HIV incidence and prevalence among female sex workers and men who have sex with men in Nairobi, Kenya?
2. Has the program successfully met its targets related to biomedical, behavioral, and structural outcomes among female sex workers and men who have sex with men in Nairobi, Kenya?
3. What is the estimated program coverage of the prevention services among female sex workers and men who have sex with men in Nairobi, Kenya?
4. What are the key barriers experienced by female sex workers and men who have sex with men when accessing and using prevention services in Nairobi, Kenya?

### Outcomes for Measurement

In total, 12 outcomes will be measured to address the study’s research questions (Textbox 1). Specifically, to address research questions 1 and 2, five biomedical outcomes, 2 behavioral outcomes, and 2 structural outcomes will be measured. For research questions 3 and 4, program coverage, availability coverage, contact coverage, and utilization coverage will be measured among both key population groups, following the definitions outlined in the program science–informed effective program coverage framework [16]. HIV prevalence and incidence at the population level will also be measured.

**Measured HIV outcomes among female sex workers and men who have sex with men using an expanded polling booth survey in Nairobi County, Kenya, from April to May 2023.**

**Themes and outcomes**

- Biomedical
  Prevalence of sexually transmitted infections (STIs) symptoms, diagnosis, and treatment
  Consistent condom use
  Routine HIV testing (and receipt of results)
  Linkage to and retention in HIV treatment and care
  HIV viral load suppression
- Behavioral
  Self-reported HIV risk behaviors
  Knowledge of HIV
- Structural
  Prevalence of gender-based violence
  Prevalence of stigma and discrimination
- Program coverage
  Availability coverage
  Contact coverage
  Utilization coverage

### Study Timeline

The ePBS study was designed with a quick turnaround; all activities are expected to be completed within 9 months. The data collection phase was planned to span 30 consecutive days, followed by data cleaning and analyses. Results will be finalized for dissemination at the end of the 9-month period.

### Study Team

#### Composition

A total of 4 study teams were established to manage the implementation of ePBS, and each team concurrently collected data in 4 different locations daily. Each team consisted of 6 members including a prevention officer, a key population outreach worker (a member of either key population community), a clinical officer, an HIV and testing service (HTS) counselor, and 2 qualitative researchers (1 focus group discussion [FGD] facilitator and 1 notetaker). Peer educators working in the SWOP clinics supported the study teams in participant enrollment. All ePBS team members, except the qualitative researchers, were enrolled from PHDA-SWOP clinic staff. All 4 teams were supervised by senior members (site supervisors) of the PHDA-SWOP clinic team and representatives from the Nairobi County government.

#### Study Team Training

The study team underwent a comprehensive 5-day training. The first 3 days of training included classroom instruction, during which the study’s investigators introduced and facilitated training on (1) the conceptual and theoretical underpinnings of the study (ie, the program science approach and the effective program coverage framework) [16]; (2) the ePBS methodology; (3) data collection methods and tools; (4) logistics, implementation flow, and study procedures; and (5) data entry processes. Subsequently, 2 days were dedicated to field practice during which mock data collection exercises were conducted to enhance the skills of the study team and to provide opportunities for troubleshooting any anticipated challenges in the field. Importantly, all 5 days of ePBS training prioritized practical exercises to ensure that the study team felt confident and proficient, thereby ensuring consistency and quality in data collection.

### ePBS Method

The ePBS method is a rapid, cross-sectional study design using complementary quantitative and qualitative methods for data collection. The innovative approach expands upon the traditional polling booth survey (PBS) method [19] by incorporating additional qualitative, quantitative, and biological data collection methods and an improved population-based approach to random sampling. Within the ePBS method, quantitative methods include (1) PBS, a group interview method in which individuals provide anonymous and unlinked responses to survey questions through a ballot, which has been shown to reduce reporting and social desirability biases for sensitive questions about conventionally stigmatizing behaviors, experiences, and social circumstances [19,20] and (2) behavioral and biological survey (BBS), which includes a short face-to-face, individual interview linked to the collection of biological samples (blood and urine). Qualitative methods include FGD with a subset of ePBS participants to inquire about factors that contribute to gaps in program coverage. Hence, the expansion of the traditional PBS by adding a BBS and FGD is referred to as ePBS.

Cross-sectional designs are commonly used for population-based surveys and to assess the prevalence of diseases in a population [21]. They are also useful for informing planning, monitoring, and evaluation of public health programs and interventions. In this study, we use quantitative methods to measure prevention program outputs and relevant outcomes among female sex workers and men who have sex with men participants, as outlined in research questions 1 to 3. We use qualitative methods to gain insight into the barriers affecting the accessibility and use of HIV services, as specified in research question 4.

### PBS Component

#### Overview

The PBS method is a well-established group survey method comprising only binary questions, in which individuals provide responses to survey questions by placing a token corresponding with their answer into a ballot box [22]. Individual responses remain anonymous and unlinked, ensuring confidentiality. This anonymity has been shown to enhance participants’ trust in the confidentiality of the data collection process, thereby minimizing social desirability biases and lending itself to more accurate reporting on sensitive behavioral information [23]. Data generated through PBS have been found to be comparable to the findings from other studies that used different methodologies with the same populations [20].

#### Sampling Procedure

Potential participants are selected using a 2-stage, randomized, probability-based sampling procedure and organized into small homogenous groups by key population groups (ie, female sex workers and men who have sex with men) and typology of the location at which sex work and cruising take place (eg, bar, bar with lodges, street, and clubs). Each PBS session consists of 10 to 12 participants of the same key population group who congregate and solicit clients or cruise in the same locations. Given the group interview format, the number of survey questions is intentionally limited, and each question is short and simple to facilitate easy response [22]. The key population program in Kenya has previously used PBS, and participants from different key population groups have found it to be an acceptable approach [7].

#### Implementation Procedure

The PBS component of the ePBS study was led by prevention officers and outreach workers. Conducting PBS requires materials that are not typically considered for other survey methods. First, each participant in a PBS session must have 3 different colored ballot boxes associated with yes, no, and not applicable responses (typically green, red, and white, respectively) and a set of cards to use as tokens to be dropped into the ballot boxes. Cards should be clearly numbered in alignment with the number of survey questions. Each participant’s station also requires a polling booth carton to create a private space that is not visible to other participants in the PBS session (often a cardboard box with 1 open face pointed at the participant). The facilitator at each PBS session should be equipped with copies of the standardized data collection tool (survey) and reporting forms to tally and record responses to each question.

Peer educators from SWOP mobilized 10 to 12 potential participants and invited them to participate in a PBS session, each of which was conducted as follows:

- Participants invited to the PBS were provided with an individual polling booth carton at the study venue. The polling booths were separated by at least 1 m to ensure privacy and confidentiality for each participant and their responses.
- The facilitator welcomed the participants and explained the objective of the study, read out the consent format, provided time and space for questions, and sought informed consent from every participant.
- Each participant received 3 ballot boxes (green, red, and white) and a pack of cards, with each card numbered and stacked in a sequential order to correspond with the number of questions in the survey.
- The facilitator confirmed that each participant had the correct number of cards arranged in the proper order before proceeding with the questions.
- The facilitator provided a full explanation of the PBS procedure, including an example question and a practice response, to assure participants that their responses will remain anonymous and unlinked.
- In terms of responses, the facilitator explained the following:
  If the response to the question is yes, the participant should put the card with the corresponding question number into the green box.
  If the response to the question is no, the participant should put the card with the corresponding question number into the red box.
  If the question does not apply to the participant, the participant should put the card with the corresponding question number into the white box.
  If the person does not want to answer the question, the card with the corresponding question number should be kept outside the provided boxes.
- The facilitator read the survey questions, one at a time, making the exercise engaging and lively. They ensured that each question was clearly understood by all participants by (1) reading each question clearly, slowly, and loudly to ensure that each participant heard the question clearly; (2) reading out the questions in a language that is easily understood by participants; (3) repeating the question, if necessary; (4) using local terms and giving sufficient pause, taking care not to rush through the questions.
- After all the PBS questions had been administered, the facilitator (1) collected the cards separately for each of the boxes: green, red, and white and (2) tallied and recorded the number of responses from each card in each of the colored boxes into a standard reporting form.
- All data generated through the PBS process were entered into a tablet by the study team using a standardized database created specifically for the ePBS. The data collection was facilitated using SurveyCTO (Dobility, Inc), a secure and scalable mobile data collection platform designed for researchers working in offline settings.

### BBS Component

#### Overview

The BBS is an expansion to the standard PBS method and was led by the study teams’ clinical officers and HTS counselors. It comprised 2 data collection methods: individual, structured interviews and biological sample collection (Table 1). Importantly, in the context of this study’s objectives and research questions, data from the BBS provide critical information to describe treatment and program coverage outcomes for the study population. BBS data are only linkable to PBS data at the group level.

**Table 1:** Biological samples collected from female sex workers and men who have sex with men during the biobehavioral survey in Nairobi, Kenya, from April to May 2023.

Biomarkers	Sample type	Type of test	Test location
HIV diagnosis	Capillary blood	Rapid test	Study site
Recency of HIV infection	Plasma	Laboratory-based test	PHDA^a^ laboratory
HIV-1 RNA viral load	Plasma	Laboratory-based test	PHDA laboratory
Actively taking PrEP^b^	Urine	Rapid test	Study site or in the PHDA laboratory

^a^PHDA: Partners for Health and Development in Africa.

^b^PrEP: pre-exposure prophylaxis.

#### Individual, Face-to-Face Structured Interviews

Following the PBS, all participants who provided informed consent participated in a brief, face-to-face, structured interview with a clinical officer to gather limited demographic information, basic behavioral information related to HIV risk, and program access and service use data. These data will be linked, at the individual level, to biological samples (blood and urine), which were collected following the face-to-face interviews.

#### Biological Samples

##### Urine Sample

To protect the confidentiality of participants’ HIV status, urine samples were collected from all participants who provided informed consent to providing biological samples. However, only samples from those participants who had reported currently taking PrEP in the preceding face-to-face interview underwent testing using a rapid tenofovir assay developed and validated by the University of California, San Francisco in conjunction with Abbott Laboratories [24]. Depending on timing and the facilities available on-site, the samples were either tested for tenofovir at the study site or sent to the PHDA laboratory for processing the following day (Table 1).

##### Blood Samples

HTS counselors collected fingerprick blood samples from all participants who provided informed consent for on-site rapid HIV testing. Following Kenyan rapid testing algorithms, Determine HIV-1 or HIV-2 (Abbott Diagnostic Medical Co Ltd) was used as the initial screening assay. All reactive samples underwent confirmatory testing using First Response (Premier Medical Corp Ltd) rapid tests. Pre- and posttest counseling sessions were administered to all participants undergoing HIV rapid testing in accordance with the national guidelines [25].

In addition, clinical officers drew 5 mL venous blood samples from consenting participants who tested positive for HIV in the rapid test. Whole blood samples were transferred to the PHDA laboratory for further analyses, including HIV viral load and recency testing (Table 1). The HIV viral load analyses will be conducted using GeneXpert HIV-1 Viral Load Kits (Cepheid). HIV recency will be determined using the Asante HIV-1 Rapid Recency Assay (Sedia Biosciences Corporation). All laboratory-based tests will be carried out in accordance with the PHDA laboratory’s standard operating procedures and in adherence to the national guidelines [26].

##### Biological Sample Management

All specimens, both urine and blood, were affixed with preprinted standard codes along with the corresponding survey identification at the time of collection. Following collection, all biological samples were carefully placed in boxes at room temperature. These samples were transported to the PHDA laboratory using PHDA vehicles for processing and subsequent testing. The transfer of samples from study sites to the PHDA laboratory followed a protocol outlined by the PHDA laboratory’s standard operating procedures. Both urine and blood samples will be maintained at a temperature of −80°C for 5 years within the PHDA laboratory per standard operating procedures. This extended storage period ensures the availability of samples for the confirmation of any discrepant laboratory results that may arise over time. Stored specimens will not be linked to any personal identifiers and cannot be traced back to the individuals who provided them, preserving the confidentiality and privacy of the participants.

### FGD Component

The FGD component of the ePBS study was led by the study teams’ qualitative researchers. For each key population group, participants were selected from every fifth PBS session and were engaged in the FGD after providing informed consent. Each FGD consisted of the same 10 to 12 participants who participated in the preceding PBS session. The FGDs were facilitated by trained qualitative researchers using an FGD guide. The discussions were conducted in either Kiswahili or Sheng, which is a local slang language predominantly spoken in urban areas of Kenya, particularly Nairobi. All FGDs were audio recorded and will be translated and transcribed verbatim for analysis.

### Participant Inclusion Criteria

To be eligible for participation in this ePBS study, female sex workers had to be assigned female at birth, aged at least 18 years, and acknowledge having received money or gifts in exchange for sexual intercourse with an individual assigned male at birth at least once in the past 3 months. Eligible men who have sex with men had to be assigned male at birth, aged at least 18 years, and report at least 1 anal sex act (insertive or receptive) with another individual assigned male at birth in the past 3 months. To be eligible, all participants had to be capable and willing to provide written or verbal informed consent to participate in the 3 components of the ePBS study, self-identify as a sex worker or a man who has sex with other men, and report actively practicing sex work or cruising within Nairobi County.

### Target Sample Size Calculation

To be representative of each key population group, the study’s sampling strategy was based on probability sampling techniques that provided an equal chance to all members of the study populations, meeting defined inclusion criteria, to be included in the study. The determination of an appropriate sample size for a single study domain is usually based on the following considerations: (1) the number of measurement units in the population, (2) the parameter of interest, and (3) the degree of confidence by which the parameter is estimated and the desired level of precision.

One objective of our study was to estimate the HIV prevalence and incidence among female sex workers and men who have sex with men in Nairobi County. As such, sampling parameters were set with the prevalence and sample size having 95% statistical confidence, with a desired precision of +5% or –5%.

The sample size for PBS and BBS study was calculated using the following formula: *n* = [1.96^2^*p*(1 – *p*)(*DEFF*)]/*d*^2^. Where *p* is HIV prevalence, *DEFF* is the design effect of the sampling approach, and *d* is the desired precision.

The finite population correction factor is then applied to the derived sample size using the following formula: *nf* = (n * *N*)/[(*n* + (*n* – 1)]. Where *nf* is the sample size adjusted for finite population correction, *n* is the sample size required, and *N* is the population size.

To calculate the sample size for this ePBS study, we used the most recent estimate of HIV prevalence among female sex workers (26%) and men who have sex with men (12%) in Nairobi, as reported in a 2017 study conducted by NASCOP [7]. The sample size calculation was adjusted to accommodate an anticipated nonresponse rate of 15%. The final target sample size, adjusted for population size and nonresponse, for female sex workers was n=769 and for men who have sex with men was n=420. Ideally, each PBS session comprises 10 to 12 participants; the total target sample sizes for female sex workers and men who have sex with men were divided by 12 to determine the total number of PBS sessions required for each key population group, that is, 64 PBS sessions with female sex workers and 35 sessions with men who have sex with men.

For each key population group, participants in every fifth PBS session were invited to participate in an FGD. In total, 20 FGDs were conducted with ePBS participants (female sex workers: *n*=13, 65%; men who have sex with men: n=7, 35%).

### Developing a Sampling Frame

#### Overview

The study used a 2-stage, random sampling approach. In the first stage, random locations (ie, places where female sex workers solicit clients or engage in sex and where men who have sex with men cruise or engage in sex) were selected from a previously validated list. In the second stage, participants were randomly selected from the locations on a randomly selected day of the week.

#### Stage 1 Sampling

As a first step, the ePBS study team developed the sampling frame by validating lists of mapped locations in Nairobi County where female sex workers and men who have sex with men solicit clients, cruise, meet sexual partners, and engage in sexual activities. The sample frame was derived from a programmatic mapping exercise conducted in 2018 [6]. The lists were updated—that is, closed locations were removed and newly emerged locations were added—through a validation process facilitated by the study team members who are in regular, direct contact with key population members. Through this validation process, population size estimates for each location, the days and times when these locations are operational, and the days when the locations have the highest number of female sex workers or men who have sex with men were verified and revised, as needed, via a standardized approach.

The lists of validated locations (1 each for female sex workers and men who have sex with men) formed the sampling frames representing the universe of locations across Nairobi County. Key population members spending time within a given location tend to be more homogeneous, in terms of typology and risk behavior, than those in different locations [27]. As such, potential participants for each PBS session were randomly selected from a single location. During the first stage of sampling, 99 locations (equal to the number of PBS sessions planned for each group) were randomly selected such that 1 PBS session was conducted per selected location. The random selection of key population group–specific locations from which potential participants were identified was stratified by subcounty and location typology.

#### Stage 2 Sampling

In stage 2 of sampling, each of the 99 randomly selected locations was randomly allocated to an assigned week and day on which potential participants were enrolled from the location. For example, data collection for this ePBS study was projected to take 30 days; for each location, 1 week (ie, from weeks 1 to 4 of the study period) was randomly selected first, followed by the random selection of 1 specific day within that chosen week. This process determined the precise week and day that each selected location was designated for the study. Ultimately, 10 to 12 potential participants were randomly approached by community researchers at the location on the specified day and week and invited to take part in the study.

This 2-stage sampling approach ensured that participants were representative of the total population of the key population group by geography (subcounty) and location typology across days of the week, yielding a comprehensive and diverse sample of participants from communities of female sex workers and men who have sex with men in Nairobi County.

### Study Procedure

**Figure 1:**
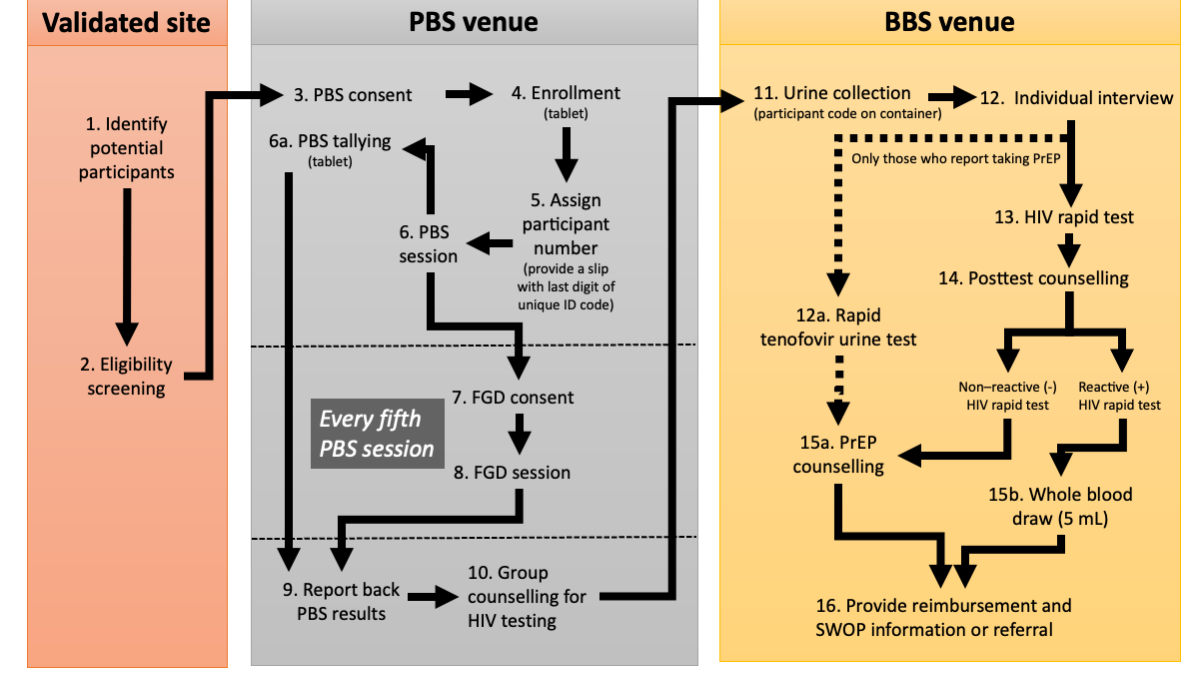
Expanded PBS study procedure flowchart used to collect data from female sex workers and men who have sex with men in Nairobi, Kenya, from April to May 2023. BBS: behavioral and biological survey; FGD: focus group discussion; PBS: polling booth survey; PrEP: pre-exposure prophylaxis; SWOP: Sex Workers Outreach Program.

The overall flow of the ePBS study procedure from participant identification and enrollment to PBS, BBS, and FGD (every fifth PBS session) is outlined in Figure 1. The duration to complete PBS and BBS was 120 minutes, including 10 minutes for initial screening and consent, 60 minutes for the PBS, 20 minutes for pretest counseling, and 30 minutes for BBS. For every fifth PBS group participating in an FGD, an additional 45 to 60 minutes was added to their total estimated participation time.

### Participant Enrollment and Data Collection Site Preparation

Potential participant enrollment occurred at selected locations on the allocated days of the week, according to the study’s sampling frame. Enrollment primarily occurred in the late afternoon or evening on the day of data collection to increase the likelihood of meeting a higher number of female sex workers or men who have sex with men in the location. Peer educators and outreach workers from the SWOP clinics facilitated the identification of potential participants within their own key population group. They were responsible for introducing potential participants to the study and assessing their eligibility. Any eligible individuals were provided with detailed information about ePBS. If eligible participants were interested in participating in the study, they were brought to the data collection site by peer educators and outreach workers. As much as possible, data collection occurred within the selected locations. Ahead of participant enrollment, the study team members scouted selected sites to identify suitable spaces for safe and confidential data collection. Team members were responsible for setting up stations for PBS and private, quiet locations for face-to-face structured interviews and biological sample collection. If the selected sites posed challenges (eg, logistical or safety challenges) for data collection, prescouted alternate locations nearby were used instead.

### Ethical Considerations

#### Informed Consent

At the data collection site, before ePBS data collection commenced, prevention officers, with the support of community researchers, guided participants through the informed consent process, ensuring that all participants understood and were comfortable with the information included in the consent form. Any questions from potential participants were addressed before commencing the study. Nonconsenting or unwilling participants were thanked for their time and excluded from the study. All eligible participants who provided written or verbal informed consent received a unique identifying code that was used for the BBS portion of the study.

#### Participant Compensation

Participants received compensation for their time and travel at a standard local rate of Ksh 500 (US $5). Those participating in FGDs received an additional Ksh 500 (US $5) for their extra time.

#### Safety

Conducting research studies with key populations who are criminalized in Kenya requires careful consideration to ensure community safety. Before initiating data collection, extensive consultations with key population communities were conducted. The primary objectives of the study and the methodology were shared and explained during these consultations. Feedback obtained from the community was instrumental in shaping the study procedures to ensure the communities’ safety and comfort. Furthermore, a collaborative partnership with Nairobi County authorities was established to enhance the safety and protection of both the study teams and the key populations and participants. The study teams carried official identity cards and study approval letters to show that they were conducting an official study. The data collection took place in locations where female sex workers and men who have sex with men generally congregate and that were considered to be safe. Location managers were sensitized and informed about the study ahead of implementation. Study team members were trained in crisis management and were provided a protocol to follow in case of an emergency or a breach in safety for the study team or participants. A team of supervisors, including senior team members of PHDA and Nairobi County officials, visited the sites each day for supervision and support.

#### Ethics Approvals

The research study has received ethics approvals from Amref Health Africa Ethics and Scientific Review Committee, Kenya (Amref ESRC P1365/2022); Health Research Ethics Board, University of Manitoba, Canada (HS25883); National Commission for Science, Technology, and Innovation, Kenya (NACOSTI/P/23/24009); and Nairobi City County, County Health Research Ethics Committee (NCCG/HWN/REC/349).

#### Privacy and Confidentiality

All investigators have completed a web-based course on the protection of human participants in research and have obtained certificates. Furthermore, investigators and all study team members have signed data confidentiality agreements with PHDA, pledging to uphold the highest standards of data privacy and security.

During the informed consent process, female sex workers and men who have sex with men participating in the study were clearly informed of their right to withdraw from the study at any point if they so desired. This withdrawal did not affect their access to the services provided by SWOP clinics. The potential benefits and risks associated with participating in the study were thoroughly explained to the participants during the informed consent process.

### Study Instruments

Standardized instruments adapted for female sex workers and men who have sex with men were used for the PBS and BBS. The FGD guide was standardized for all populations. All tools were available in English and Kiswahili.

### Data Entry and Management

Each study team had 2 tablets that were preloaded with customized web-based survey software (SurveyCTO) and were used for data entry and management. Data collected during PBS sessions were first compiled in hard copy format using standardized reporting forms that were filled out by the prevention officer and community researchers after PBS responses were tallied. Reporting form tallies were then entered into tablets by the prevention officers on each study team. BBS data were directly entered into tablets using SurveyCTO. Data quality checks were automated within the web-based system. Data were uploaded securely to a central server at the end of each field day via a virtual private network (VPN).

Select biological samples collected were sent to the PHDA laboratory (Table 1). These laboratory data were managed using the Laboratory Information Management Software (LMIS), uploaded to a repository in CSV format, and merged with data collected through SurveyCTO by the study’s data manager.

The study team’s qualitative researchers were responsible for digitally audio recording FGDs and transcribing and translating (into English) the FGD data.

### Data Security

Data were transmitted over an encrypted channel (HTTP secure), and personal identifiers were automatically encrypted at data entry. Access to central servers is restricted. Data will be stored in encrypted format for 5 years, following national data security protocols.

### Data Cleaning

Daily data quality checks will be performed on PBS and BBS data collected through SurveyCTO, addressing discrepancies, missing data, and inconsistencies as they are identified. The study teams’ prevention officers and site supervisors will oversee the data cleaning and quality assurance procedures.

### Data Analysis

Data from PBS and BBS will be analyzed at the group and individual levels, respectively. Descriptive statistics will be calculated, and univariate analyses will be conducted to measure previously specified outcomes (Textbox 1) and assess risk factors associated with HIV. Guided by the coverage cascade element of the effective program coverage framework [16], coverage gap analyses will be performed to identify and quantify gaps in availability coverage, contact coverage, and use coverage in relation to required coverage targets.

Thematic analysis will be done for FGD data to capture recurring concepts and ideas related to barriers and facilitators to accessing, contacting, and using prevention services that emerged during discussions.

### Knowledge Translation and Dissemination

The study’s findings and lessons on the feasibility and utility of ePBS as a data collection method will be shared through participation in the Nairobi County Key Population Technical Working Group and the National Key Population Technical Working Group led by the NASCOP. This working group comprises members representing implementing partners, key population community representatives, donors, and the Kenyan Ministry of Health officials. These stakeholders will have the opportunity to use the study’s findings to inform and strengthen programs and policies for key populations in Nairobi. In addition, the study results will be shared with the PHDA-SWOP Community Advisory Board and peer educators. These insights will support the development of strategies to enhance program effectiveness and improvement.

Research findings and methodological insights from implementing the novel ePBS study will be shared with academic communities in Kenya and globally through international conferences and peer-reviewed journal articles. PHDA is a part of an extensive, existing network of researchers in public health sciences, health policy, and basic and natural sciences who focus on HIV and sexually transmitted infections. Other scientific forums within the University of Manitoba will be leveraged to share the findings.

## Results

The study enrolled 759 female sex workers (response rate: 759/769, 98.6%) and 398 men who have sex with men (response rate: 398/420, 94.7%) across 64 and 35 PBS sessions, respectively (Table 2).

In total, 758 (99.9%) female sex workers (1 participant refused participation in BBS) and 398 (100%) men who have sex with men participated in the face-to-face interview for the BBS, and all (n=1156) provided blood and urine samples for rapid tests. In total, 14.79% (171/1156) of participants (female sex workers: n=101, 59.1%; men who have sex with men: n=70, 40.9%) tested positive for HIV via rapid test. Among those testing positive, 93.6% (160/171) of participants (female sex workers: n=100, 62.5%; men who have sex with men: n=60, 37.5%) provided consent for venous blood sample collection. Three whole blood samples from female sex worker participants were discarded due to missing bar codes on the sample tubes. A total of 73 participants reported currently receiving PrEP in the BBS face-to-face interview (Table 2), and rapid urine tests were conducted on the samples from these participants (female sex workers: n=56, 77%; men who have sex with men: n=17, 23%).

**Table 2:** Expanded polling booth survey (ePBS) participant enrollment and data collection among female sex workers and men who have sex with men in Nairobi County, Kenya, from April to May 2023 (n=1157).

	Female sex workers (n=759), n (%)	Men who have sex with men (n=398), n (%)
PBS participants	759 (100)	398 (100)
BBS^a^ participants	758 (99.9)	398 (100)
HIV rapid tests conducted	758 (99.9)	398 (100)
Reactive HIV rapid test results	101 (13.3)	70 (17.6)
Venous blood samples collected	100 (13.2)	60 (15.1)
Urine tests conducted	56 (7.4)	17 (4.3)

^a^BBS: behavioral and biological survey.

A total of 20 FGDs were completed with female sex workers (n=14, 70%) and men who have sex with men (n=6, 30%).

Data cleaning and analysis are ongoing. Aggregate responses from each PBS session and individual data from BBS respondents have been entered into separate data modules in the data entry software. The data will then be exported to SPSS (version 28.0; IBM Corp) for analysis. Weights will be applied to data during analyses to account for the sampling design and to adjust for unequal selection probabilities. CIs will be calculated, and descriptive analyses of the PBS and BBS data will be performed separately to address the study objectives.

The FGD data will be simultaneously translated and transcribed into English by the qualitative study team. Qualitative notes taken during the FGDs will be incorporated into a final transcript to provide appropriate contexts and details where necessary. Thematic analysis will be used to systematically capture recurring concepts, ideas, and topics that emerged during the discussions.

Furthermore, we will use the effective program coverage framework [16] to assess gaps in availability coverage, contact coverage, and use coverage within the condom, PrEP, and antiretroviral therapy program components. Equity analyses will be conducted with program coverage data to assess inequalities in the coverage of each component intervention based on age, geography, and location typology.

## Discussion

### Overview

Joint United Nations Programme on HIV and AIDS encourages countries to annually measure outcomes using nimble, rapid, and low-cost methods [28]. Through this study, the novel ePBS method was implemented for the first time, which expands upon the traditional PBS method by integrating BBS and FGD modules with minimal requirements for additional resources. Furthermore, this innovative approach adopted robust sampling methods to ensure the representativeness required for a population-based survey, unlike the traditional PBS method, which uses program-based sampling approaches [7,23]. This approach included data gathered at the group level (PBS and FGD) and individual level (BBS), encompassing behavioral, biological, and structural aspects of a combination prevention approach, from a total sample of 1157 participants within a 30-day timeframe. This method significantly enhances the range of tools currently available to collect routine outcome data from HIV prevention programs. Importantly, the ePBS method empowered the research team to efficiently gather a rich data set comprising both quantitative and qualitative data for program optimization and expanded the understanding of local HIV epidemics among key populations. The inclusion of community researchers in the study team and the involvement of peer educators at the sampled locations fostered trust and instilled confidence among study participants, enhancing the study’s credibility, which is reflected in the study’s high response rate.

### Program and Policy Implications

Notably, this study draws upon a program science approach using the effective program coverage framework to assess HIV program coverage [16,18] and outcomes among female sex workers and men who have sex with men in Nairobi County, Kenya. This embedded approach facilitates the emergence of research questions from programmatic needs and challenges, and findings contribute to defining program and policy priorities. The study questions were formulated by PHDA-SWOP in collaboration with the community advisory board and the Ministry of Health, Nairobi County, who are the primary beneficiaries of the study findings. Engaging stakeholders involved in implementing key population programs, advocating for their rights, developing policies, and being accountable for creating an enabling environment enhance their commitment to use the study’s findings [29].

The study will provide comprehensive data on biological, behavioral, and structural outcomes, as well as program coverage, within an HIV prevention program tailored for female sex workers and men who have sex with men in Nairobi County. In alignment with the Global AIDS Strategy 2021 to 2026, which includes a high-level target of ensuring that 95% of people at risk of HIV infection have access to and use effective combination prevention options, our study findings will serve as a critical measure of the outcomes of such a combination prevention program [30]. In resource-limited settings, the use of ePBS methods can serve as a pragmatic and affordable approach to assessing program outcomes. It allows for rapid data collection and accommodates the integration of qualitative data collection methods and individualized surveys with standard PBS. Critical to a program science approach, because this research is embedded within Nairobi County’s key population program, the results of this ePBS study can be seamlessly incorporated to refine the program strategy to improve coverage and outcomes. In addition, this embeddedness allowed for the ePBS study to be carried out by the program team, thereby minimizing expenses in comparison to traditional integrated biobehavioral surveillance surveys.

### Limitations

It is important to acknowledge that this study has inherent limitations, primarily due to its cross-sectional design. The results of this study will be descriptive in nature and will not contribute to the understanding of causal relationships. In addition, the study relies upon several self-reported variables, which are always subject to recall and social desirability biases. Finally, this study, and the ePBS method generally, greatly benefit, in terms of scalability and implementation, from being embedded within an established HIV prevention program for key populations, which might not be feasible in all settings.

### Conclusions

The ePBS method has the potential to meet the demand for a rapid, cost-effective, and practical approach to regularly measuring HIV outcomes in key population programs and generating insights for ongoing program improvement. The study gathered crucial data on HIV outcomes and piloted a new method called ePBS, which combines PBS, BBS, and FGDs to enhance its practicality. Our findings will help improve the HIV prevention program for key populations and develop policies in Nairobi County.

## Abbreviations

BBS: behavioral and biological survey
ePBS: expanded polling booth survey
FGD: focus group discussion
HTS: HIV and testing service
LGBTQI+: lesbian, gay, bisexual, transgender, queer, intersex, and others
NASCOP: National AIDS and STI Control Programme
PBS: polling booth survey
PHDA: Partners for Health and Development in Africa
PrEP: pre-exposure prophylaxis
SWOP: Sex Workers Outreach Program

## Data Availability

The data sets generated and analyzed during this study will not be publicly available due to the sensitivity of the data collected—sex work and same-sex relationships are criminalized in Kenya—and a prevailing antagonistic environment against LGBTQI+ (lesbian, gay, bisexual, transgender, queer, intersex, and others) populations exists across the country. However, data can be made available from the corresponding author on reasonable request.
